# Medicinal Liquid Vaseline

**Published:** 1887-11

**Authors:** 


### MEDICINAL LIQUID VASELINE.

### A NEW VEHICLE FOR HYPODERMIC INJECTIONS.

Hypodermic medicine seems likely to realize an immense extension in the use, as an excipient for medicinal substances which it is desired to introduce subcutaneously, of a petroleum product which comes from Europe, and especially from the mountainous regions of Russia, and is known as *liquid vaseline.* This is one of the final products of the distillation of petroleum—coming over after paraffine—and is purified by filtering through animal charcoal. Its solvent powers are immense, multitudes of vegetable and mineral substances readily dissolving in it, and what renders this oil especially valuable is that it is not only unirritating of itself, but it so sheathes and modifies medicaments of a locally irritant character (rendering them bland) as to constitute them suitable and safe for hypodermic use.

Among the substances soluble in liquid vaseline, we may enumerate eucalyptol, iodoform, turpentine, phenol, thymol, iodine, menthol, chloroform, ether, salol, sulphide of carbon, sulphuretted hydrogen, myrtol, cocaine, atropine, aconitia, caffeine, calomel, phosphorus, bromine, etc. Dr. Albin Meunier has most patiently and successfully worked out the requisite formulæ for hypodermic injections with liquid vaseline for their basis. Among these formulæ none seems to have given better satisfaction in pulmonary and bronchial complaints than the combination of eucalyptol with liquid vaseline—one part of the former to five of the latter. Five cubic centimeters of this solution are injected into the gluteal region night and morning, and under this treatment has been noticed a marked lessening of the cough and expectoration. A two-per cent. solution of iodine in liquid vaseline is highly spoken of in asthma, emphysema, the uric acid diathesis, etc.; the dose is one syringeful, to be injected into the outer part of the thigh. A twenty-per-cent. solution of chloroform is recommended to be injected in sciatica and other painful affections, and a ten-per-cent. solution of salol is well spoken of in sciatica. The injections must be made deeply in the muscles. There are formulæ for bromine for antisepsis in diphtheria, and calomel for administration in syphilis.

Bourneville and Bricon have recently published a series of convenient formulæ, with liquid vaseline for basis, in their therapeutic manual, and the members of the Sociètè de Thèrapeutique seem enthusiastic in their praises of the new mode of treatment, which bids fair greatly to add to our therapeutic resources by enabling us to use hypodermically multitudes of remedies which heretofore could not be administered in this way.

Liquid vaseline, according to Roymond, is the result of the depuration of the mineral oil of Baccou in Russia. In distilling this oil, several intermediate substances first come over (ethers and illuminating oils), finally paraffine and liquid vaseline. The Germans call the latter *liquid paraffine.*—*Boston Medical and Surgical Journal.*

